# Metabolomic phenotyping reveals the GxExS blind spot in berry fruit quality breeding

**DOI:** 10.3389/fpls.2026.1874967

**Published:** 2026-07-01

**Authors:** Patricia Pacheco-Ruiz, Sara Postacchini, Bruno Mezzetti, Luca Mazzoni, Sonia Osorio, José G. Vallarino

**Affiliations:** 1 Instituto de Hortofruticultura Subtropical y Mediterránea “La Mayora”, Departamento de Biología Molecular y Bioquímica, Universidad de Málaga-Consejo Superior de Investigaciones Científicas (IHSM-UMA-CSIC), Málaga, Spain; 2 Department of Agricultural, Food and Environmental Sciences (D3A), Università Politecnica delle Marche, Ancona, Italy

**Keywords:** blueberry, genomic selection, GxExS interactions, metabolic selection, metabolic stability, raspberry, strawberry

## Abstract

Genomic selection (GS) has accelerated fruit quality improvement in strawberry, blueberry, and raspberry breeding, yet it operates under the assumption that genetic effects on quality metabolites are stable across environments and seasons. Recent metabolomics evidence challenges this assumption, revealing that Genotype × Environment × Season (GxExS) interactions can reshape metabolic profiles and reverse genotype rankings for consumer-relevant traits. In this review, (1) we evaluate the predictive capacity of GS and metabolomic phenotyping for fruit quality across the three major berry crops, (2) identify the GxExS gap as a critical barrier to the transferability of these predictions to field applications, and (3) propose multi-environment metabolomic evaluation as the missing layer to bridge this gap. Studies in blueberry have demonstrated that metabolomic and phenomic selection achieve comparable predictive accuracy to GS for quality traits, and that metabolite-based models can predict consumer liking. Yet these frameworks have been developed and validated predominantly within single environments, without systematic assessment of their stability across contrasting locations or years. Integrating metabolomic profiling into multi-environment breeding trials would enable the identification of genetically robust versus environmentally plastic metabolites, providing breeders with reliable selection targets for producing climate-resilient cultivars with consistent fruit quality.

## 1 Introduction

Genomic selection has reshaped berry breeding over the past decade. By correlating high-density marker data with phenotypic records, GS compresses the breeding cycle and captures additive variance for complex traits at costs that phenotype-driven selection cannot match (Lee et al., 2024; Senger et al., 2022). In strawberry (*Fragaria* × *ananassa*), blueberry (*Vaccinium corymbosum*) and raspberry (*Rubus idaeus*) — three economically important crops (Bezerra et al., 2024) — GS now delivers useful prediction accuracies for yield, firmness and shelf life (Adunola et al., 2024b; Yamamoto et al., 2021).

Fruit quality, however, refuses to behave. Flavor, aroma and nutritional composition emerge from dozens of volatile and non-volatile metabolites whose concentrations respond to ripening stage, water status, photoperiod and temperature as strongly as they respond to genotype (Pacheco-Ruiz et al., 2026). Models that predict breeding values from SNP data alone routinely fail on these traits because the phenotype the breeder wants is not written in the genome in isolation. It is written in the interaction.

Metabolomic phenotyping offers a direct read of that interaction. Targeted and untargeted profiling of primary and secondary metabolites captures the chemical state of the fruit at the moment of harvest, bypassing the genotype-to-phenotype inference entirely and feeding the downstream predictor with variables that already encode environmental history (Colantonio et al., 2022; Fan et al., 2021). For traits governed by polygenic architectures expressed on tetraploid, hexaploid or octoploid genomes, this is not a minor shift.

Yet almost every published metabolomic prediction framework for berries has been trained and validated in a single location, a single season, or both. The transferability question across contrasting environments has rarely been asked, and when asked, rarely answered with multi-site multi-season data. The Genotype × Environment × Season (GxExS) gap that this question opens is, in our reading, the operational blind spot of current berry quality breeding. This mini-review evaluates metabolomic phenotyping for fruit quality in strawberry, blueberry and raspberry, identifies the GxExS gap, and proposes a framework for integrating multi-environment metabolic stability into breeding pipelines.

## 2 Metabolomic prediction of quality: what works and where it breaks

Fruit quality is a composite trait: flavor, aroma, texture and nutritional composition arise from interacting metabolic networks whose outputs are read by the consumer as a single impression. Statistical and machine-learning models trained on metabolite data can capture that composite signal within a given program, but the predictors change with species, ploidy and the chemistry of the trait itself.

### 2.1 Blueberry

Blueberry is the crop where metabolomic selection has advanced furthest. Ferrão et al. (2020) combined genome-wide association and volatile profiling on a University of Florida panel to map candidate loci for aroma compounds, several encoding biosynthetic enzymes, and reported high marker-based predictive abilities for most volatile organic compounds (VOCs). A subsequent pedigree-plus-metabolome analysis identified monoterpenes and sesquiterpenes as the dominant drivers of floral and sweet aroma perception, with heritabilities high enough to be used as breeding targets (Ferrão et al., 2022). Ferrão et al. (2025) extended this framework using multivariate adaptive regression splines (MARS) to derive empirical thresholds — minimum concentrations at which individual compounds shift consumer perception — converting continuous metabolite data into actionable selection criteria for breeders working below, at, or above those values.

The most aggressive test of metabolomic selection to date came from Colantonio et al. (2022), who trained XGBoost and other machine-learning models on targeted VOC profiles of diverse blueberry accessions to predict consumer sensory ratings. XGBoost models trained on targeted metabolomics achieved prediction accuracies of r = 0.87 for sourness and r = 0.75 for sweetness in blueberry, and volatile organic compounds explained 56% of the variance in overall consumer liking — far more than sugars and acids combined. The result established metabolomic selection as feasible in blueberry and not as an accessory to GS but as a competitive alternative for flavor traits where markers alone underperform. Complementing these spectrometry-based approaches, Adunola et al. (2024a) showed that near-infrared spectroscopy (NIRS) of fruit and leaf tissue delivered prediction accuracies for Brix and the Brix:titratable acidity ratio comparable to those of full genomic selection in a 372-genotype panel evaluated over two years. NIRS is orders of magnitude cheaper than LC-MS or GC-MS, which matters when thousands of seedlings must be screened.

These results share a limitation the papers do not hide: training and validation populations come from a single breeding program (University of Florida, Gainesville), often a single location and one or two seasons. Whether these models retain their accuracy when transferred to a Michigan, Chilean or Huelva production environment remains an open empirical question.

### 2.2 Strawberry

Strawberry flavor is chemically denser than blueberry and genetically harder to dissect. The cultivated strawberry is an allo-octoploid with substantial subgenomic redundancy, which complicates marker-assisted prediction and makes direct metabolite phenotyping an attractive parallel route. Consumer preference consistently weights sweetness and complex aromatic notes above all other sensory descriptors (Colquhoun et al., 2012), and the chemistry behind those notes is dominated by volatile compounds whose abundance is barely correlated with sugar content.

Fan et al. (2021) identified twenty VOCs — esters, furanones and terpenoids — that enhance perceived sweetness independently of soluble solids, defining a flavor-breeding target where metabolite panels are the natural instrument.

The stability of those panels is the open question. Schwieterman et al. (2014) phenotyped a strawberry diversity panel across two seasons and reported significant between-year shifts in flavor metabolites and in their correlations with sensory ratings, the same cultivars producing measurably different profiles. Our group subsequently confirmed and extended this pattern in a multi-omics evaluation of five European cultivars grown in Poland, Germany, France and Italy across two seasons (Pacheco-Ruiz et al., 2026): a substantial fraction of primary metabolites and volatiles — sucrose, γ-decalactone, mesifurane — shifted dramatically with location and year, while a smaller subset such as linalool remained stable. Single-environment prediction frameworks for strawberry flavor are therefore working with moving targets; the metabolite that ranked a cultivar first in Poland can rank it last in Germany. That ranking reversal is not a statistical artefact. It is the breeding problem.

### 2.3 Raspberry

Evidence for raspberry is thinner, and this is itself a finding. Consumer studies identify sweetness and aroma intensity as dominant preference drivers (Hancock et al., 2018; Villamor et al., 2013) and volatilomic surveys have described the compound classes involved (Farneti et al., 2023), but few metabolite-to-sensory prediction models comparable to the blueberry or tomato literature exist. Durán-Soria et al. (2021) profiled the chemical composition of raspberry fruits across European sites and reported strong GxE structure in sugars, acids and anthocyanins, with environment accounting for variance fractions that rivalled genotype for several compounds. Willman et al. (2022) extended the signal to the genetic architecture itself, showing significant QTL-by-environment interactions for quality traits in black raspberry (*Rubus occidentalis*). The conclusion is consistent: raspberry metabolite chemistry is strongly environment-dependent, and prediction models trained in one location cannot be assumed to transfer. Recent multi-omics integration in Rubus has begun to address this gap. Sargent et al. (2025) combined metabolomics, transcriptomics and long-read genome sequencing to identify a CACTA-like transposon disrupting Anthocyanidin synthase as the genetic basis for apricot fruit color, demonstrating that multi-omics dissection of single quality traits is now feasible in raspberry. At broader scale, Zhang et al. (2025) assembled a Rubus pan-genome and integrated metabolomic and transcriptomic data across wild and cultivated species, identifying regulatory bottlenecks in anthocyanin transport. Neither study, however, evaluated multi-environment or multi-season stability, and the operational gap for metabolomic-assisted breeding in raspberry remains.

The three crops converge on the same diagnosis. Metabolomic and phenomic tools perform well as phenotyping layers and deliver competitive prediction accuracies for flavor and quality traits within a given training set. The predictions remain environment-dependent. Multi-environment trials across the three species show that the metabolic profiles carrying the predictive signal shift with location and season, and the lack of transferability is the primary bottleneck holding metabolomic selection back from routine deployment in commercial breeding programs.

## 3 The genotype × environment × season blind spot

For organoleptic and nutritional traits, that condition rarely holds. A genomic estimated breeding value describes an expectation; the metabolic phenotype of a ripe berry is that expectation filtered through the specific environment and season in which it matured. Selection on the expectation alone produces cultivars that perform as predicted in one place and fail elsewhere.

Two components of environmental variation deserve separate treatment because they impose different costs on the breeding program. Spatial GxE captures the effect of geography (soil, latitude, photoperiod, altitude, management) on performance of the same genotype across sites. This component is partially manageable: the breeder can stratify trials, match cultivars to target mega-environments and plan around known site effects with enough prior data. Temporal GxS captures the effect of year-to-year and season-within-year climatic fluctuation at the same site, under identical management. Temperature, rainfall, radiation and heat-wave timing vary between seasons in ways breeders cannot anticipate. For berry quality traits, temporal variance is often the harder problem, and it is amplified by climate change.

Recent multi-environment and multi-season data sets make the magnitude of GxExS effects on berry metabolic profiles concrete. Wang et al. (2025) phenotyped an F1 population derived from ‘Senga Sengana’ × ‘Candonga’ across five European sites spanning climates from Mediterranean to continental. Genotype emerged as the dominant source of variance for most measured metabolites, but environment and season significantly reshaped concentrations of several quality-relevant compounds, with the relative contribution of each variance component differing by metabolite class. A subset of malonylated anthocyanins inherited in a 1:1 ratio independent of trial location, with the enzymatic basis identified, exemplifies the genetically buffered class whose genetic gain transfers cleanly. Our own data in five European cultivars confirmed the same duality (Pacheco-Ruiz et al., 2026): linalool concentrations remained comparable across climates, stable within a factor of two across four European sites and two consecutive seasons, whereas sucrose, γ-decalactone, mesifurane and several other flavor-relevant metabolites varied by more than an order of magnitude between locations and seasons, with the strongest responses linked to temperature accumulation during the two weeks preceding harvest.

Quantitative variance partitioning makes these effects concrete. In our multi-omics European trial, PERMANOVA assigned 31.1% of total metabolome variance to environment, 19.5% to cultivar and 17.8% to the Genotype × Environment interaction, with harvest date and its interactions contributing below 5% (Pacheco-Ruiz et al., 2026). At the compound level, sugars in open-field conditions showed GxE effects of approximately 20% of variance, sucrose under polytunnel was driven 26% by environment, and malic acid showed 17.6% GxE in polytunnel. By contrast, linalool, mesifurane and γ-decalactone were dominated by cultivar effects with limited GxE, behaving as the robust end of the spectrum we propose. To our knowledge, no published berry study has externally validated a metabolite-based prediction model in a fully independent breeding program, confirmed by systematic literature search for the period 2020–2026; this remains a structural gap of the field that future multi-program multi-environment trials must address.

Ranking reversals are the operationally critical consequence. In our multi-site trial, the GGE Mean-versus-Stability analysis ranked ‘Clery’ as the top performer under Polish conditions and ‘Gariguette’ as the top performer under German and French conditions, with ‘Frida’ leading in Norway (Pacheco-Ruiz et al., 2026). The Multi-Trait Stability Index further identified ‘Sonata’ as the only cultivar combining high mean performance and broad environmental stability across the five sites. Selection based on any single-site evaluation would have produced different and partly contradictory recommendations. Selection of ‘Clery’ as the flavor target based on any single-site metabolomic prediction would have misled the program. In blueberry, Redpath et al. (2021) documented that harvest timing within a single season shifted firmness, acidity and soluble solids enough to change consumer perception of the same genotype. In raspberry, Durán-Soria et al. (2021) found soluble solids, total acidity and total anthocyanin content all strongly GxE-modulated across European trial sites while showing comparatively smaller GxS components, with the spatial signal therefore dominating over the temporal one for this crop in the data sets available so far.

**Figure 1:**
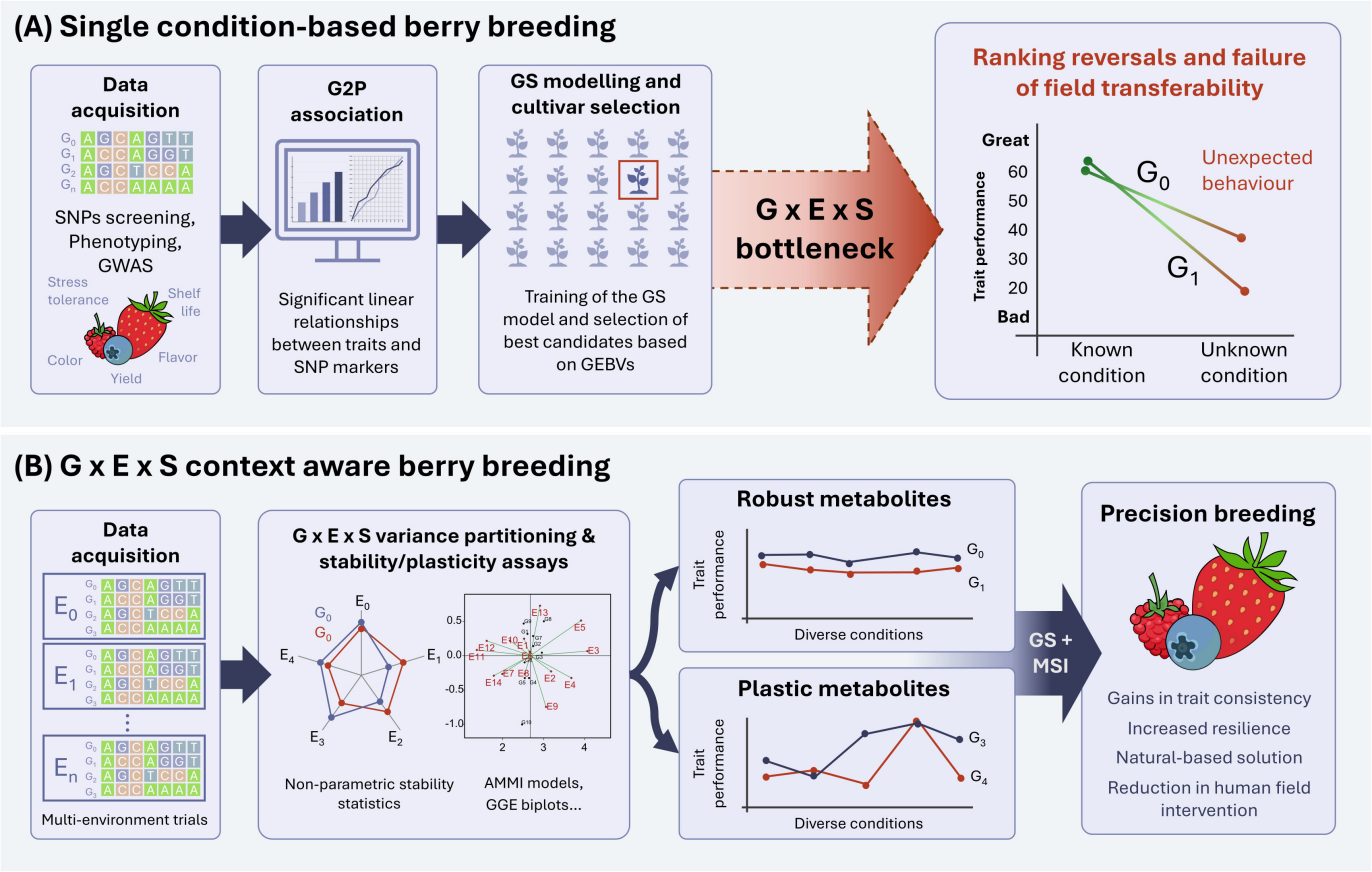
Comparison between conventional single-condition breeding and G × E × S context-aware precision breeding in berry crops. **(A)** Traditional breeding workflow based on a single environmental condition. Phenotypic and genotypic data are coupled to identify quantitative trait loci (QTLs) via genome-wide association studies (GWAS) within a single environment. This matrix informs Genomic Selection (GS) models to estimate Breeding Values (GEBVs), facilitating the selection of elite candidates. However, these models often fail to account for non-additive genetic effects or environmental interactions, leading to ranking reversals and poor field transferability in uncharacterized environments. **(B)** G × E × S context aware berry breeding. This framework captures G × E × S dynamics by integrating multi-environment trial (MET) data with high-throughput multi-omic layers. By employing multivariate models [e.g., AMMI, GGE biplots, or the metabolic stability index (MSI)], the system deconstructs phenotypic plasticity to discriminate between stable (robust) and environment-specific (plastic) metabolites.

The evidence supports an operational distinction that we propose as a framework for metabolomic selection. *Robust* metabolites — those under strong genetic control and minimal GxExS modulation within the tested range of environments — are reliable selection targets whose genetic gain transfers across locations and seasons. The malonylated anthocyanins of Wang et al. (2025) are the textbook case in strawberry; linalool appears to play a similar role for aroma, and specific terpenes in blueberry behave comparably (Ferrão et al., 2022). *Plastic* metabolites exhibit substantial GxExS sensitivity and reorder genotype rankings across sites or seasons. Sucrose in strawberry is the paradigmatic example: nutritionally relevant, sensorially important, and mechanically unreliable as a selection target without multi-environment validation. Between these two extremes lies a continuum of intermediate metabolites whose classification depends on the specific environmental range sampled and on the statistical threshold the breeder accepts for calling a metabolite stable. The distinction is therefore operational, not biological: a metabolite is robust or plastic with respect to the specific set of environments and seasons that define the target production range. Classifying metabolites into these two categories before writing the selection index — rather than averaging over the full metabolome and hoping that environmental noise cancels — is the conceptual contribution that multi-environment metabolomics brings to genomic selection for fruit quality (**Figure 1**).

Operationally, variance partitioning through AMMI, GGE biplots or Finlay-Wilkinson regression separates genotype main effects from environment, season and interaction components and yields a quantitative stability index per metabolite, implementable in R packages such as metan or sommer. Robust metabolites then enter the multi-trait selection index with full weight as primary targets, plastic metabolites are restricted to mega-environment-specific indices or excluded until reaction norms are characterized, and the metabolic stability index becomes a covariate alongside the genomic estimated breeding value.

## 4 Integrating metabolic stability into breeding pipelines

Translating the robust-plastic framework into a working breeding program requires a defined multi-omics workflow. We outline one such workflow in four stages (**Figure 1**). First, the breeding population is genotyped at high density through SNP arrays or genotyping-by-sequencing (McCallum et al., 2016), establishing the genomic scaffold on which subsequent analyses rest. Second, metabolomic profiling is performed across a set of contrasting locations and at least two seasons, sampling the range of temperature, water and radiation regimes the cultivar will eventually face (Durán-Soria et al., 2021; Wang et al., 2025). The choice between targeted LC-MS/MS panels and untargeted Orbitrap-based profiling is dictated by cost constraints and by how many metabolites the program needs to track; no single technique is obligatory.

Third, variance partitioning classifies metabolites. Additive main effects and multiplicative interaction (AMMI) analysis, genotype-plus-genotype-by-environment (GGE) biplots and Finlay-Wilkinson regression separate genotype main effects from environment, season and interaction components (Adunola et al., 2024c; Pacheco-Ruiz et al., 2026; Prohaska et al., 2024). The output is a classification of every measured metabolite as robust, plastic or intermediate within the tested environmental range, together with a quantitative stability index that can be carried into downstream selection. Fourth, selection indices are constructed. Robust metabolites receive high weights as primary selection targets and define the genetic gain that should transfer across the production area. Plastic metabolites enter as site-specific targets or are dropped from the index until their reaction norms are characterized.

The final stage combines genomic and metabolic predictors. Genotype-plus-metabolome models of the type implemented by Adunola et al. (2024a) in blueberry integrate genomic estimated breeding values with metabolic stability indices to predict multi-environment phenotypes, consistently outperforming GS alone on traits where metabolic plasticity is non-trivial. Direct empirical demonstrations of this dual-layer architecture are still rare in berry crops. In the perennial Coffea canephora — a methodological analog of long-cycle berry breeding — Adunola et al. (2023) showed that environmental stability metrics are themselves genomically predictable, allowing simultaneous selection for trait performance and environmental stability in the presence of substantial genotypic ranking crossover. The conceptual transfer to berry metabolomic selection is direct: stability indices computed from variance partitioning can enter the prediction model as an additional response variable alongside the trait itself. Robust quality phenotypes are also natural candidates for low-input production systems, aligning the breeding output with nature-based strategies.

## 5 Conclusions

Bridging the GxExS gap requires multi-environment metabolomic evaluation as a routine layer of the berry breeding pipeline, not an exploratory add-on. The evidence base reviewed here derives from a relatively limited number of breeding programs — predominantly the University of Florida program for blueberry and the GoodBerry and BreedingValue consortia for European strawberry and raspberry — which constrains the transferability of conclusions across germplasm pools, production systems and climatic regions. Four gaps remain open. The strawberry evidence for GxExS modulation of quality metabolites is the most developed of the three crops, and blueberry and raspberry require comparable multi-site, multi-season metabolomic data sets before robust-plastic classifications derived in single programs can be generalized to commercial breeding pipelines elsewhere. Untargeted metabolomics remains too expensive to deploy at the scale of a commercial breeding trial; rationalization of target panels to the 20 to 50 compounds that carry most of the sensory and nutritional signal is a practical prerequisite for adoption, and the MARS-based threshold work of Ferrão et al. (2025) illustrates how that rationalization can be made consumer-driven rather than analyst-driven. Beyond panel size, deployment at multi-program scale requires standardization of harvest stage, time-of-day sampling and post-harvest handling, adoption of community metabolite identification standards (Sumner et al., 2007), and deposition of raw spectra in public repositories such as MetaboLights and Metabolomics Workbench as minimum requirements for cross-laboratory comparability. Field-deployable phenotyping platforms are the third gap: portable NIRS systems, of the type validated by Adunola et al. (2024a), offer a realistic route to routine metabolomic phenotyping in breeding plots, bypassing the sampling-to-analysis latency that makes laboratory mass spectrometry unworkable at selection-cycle timescales. The fourth gap is environmental: current GS models for berry quality traits remain environment-naive, and explicit incorporation of ripening-period temperature, cumulative radiation and water-status covariates into prediction models is the frontier where genomic and metabolomic prediction converge. Closing these gaps would let breeders combine genetic gain with metabolic resilience and align berry breeding with the nature-based intensification demanded under climate change.
